# Research on fall detection algorithm for older adults in complex lighting environments based on improved YOLOv8n

**DOI:** 10.3389/fpubh.2026.1787316

**Published:** 2026-03-24

**Authors:** Xiaojin Gan, Yihui Lai, Gui Xiao, Yuting Xiong, Leyan Yu, Xiaoling Zhou, Songying Wu

**Affiliations:** 1 Department of Gynecology, The Affiliated Hospital of Jiangxi University of Chinese Medicine, Nanchang, Jiangxi, China; 2 School of Intelligent Medicine and Information Engineering, Jiangxi University of Chinese Medicine, Nanchang, Jiangxi, China; 3 Department of Nursing, Jiangxi University of Chinese Medicine, Nanchang, Jiangxi, China

**Keywords:** attention mechanism, fall detection, lightweight, module fusion improvement, YOLOv8n

## Abstract

**Background:**

With the continuous intensification of global aging, the issue of older adult(s) falls has become a significant public health challenge. Real-time and accurate fall detection technology is of great importance for ensuring the safety of the older adult(s). To address the problems of difficult discrimination of fall features caused by human movement in complex backgrounds and low detection accuracy, this study proposes a fall detection method for the older adult(s) based on the improved YOLOv8n, aiming to effectively identify fall behaviors of the older adult(s) in complex lighting and shadow environments.

**Methods:**

Firstly, a multi-pose human fall database containing different light intensities and diverse real-life scenarios was constructed. Secondly, based on the YOLOv8n network architecture, seven advanced lightweight and attention mechanism modules, namely the Reparameterization Visual Geometry Group (RepVGG) module, the Spatial Pyramid Pooling with Fast Large Separable Kernel Attention (SPPF_LSKA) module, the Shuffle Attention (SA) module, the C2f module with Spatial and Channel Reconstruction Convolution (C2f_ScConv), the Simplified Spatial Pyramid Pooling-Fast (SimSPPF) module, the Squeeze-and-Excitation Attention (SEAttention) module, and The C2f module with Partial Kernel Interaction (C2f_PKI), were introduced, and 20 groups of improvement experiments covering single-module, dual-module, and triple-module fusions were conducted. The impact of different improvement strategies on model performance was systematically analyzed. Finally, the optimal model was selected for field verification.

**Results:**

Experimental results demonstrate that different improvement strategies exert varying effects on the performance of the YOLOv8n model, among which the dual-module enhancement combining C2f_PKI and SimSPPF has proven to be the most effective. Under the condition of maintaining the same parameter count, this improved model achieved an mAP@0.5 of 91.8% on a self-constructed dataset, representing a 2.1% increase over the baseline YOLOv8n model, with an inference speed reaching 41.6 frames per second. The detection accuracy and efficiency of this model surpass those of mainstream models such as YOLOv5s and Faster-RCNN. Furthermore, its detection performance was validated on the publicly available UR Fall dataset, where it achieved an accuracy improvement of up to 10% in real-world scenarios.

**Conclusion:**

The study demonstrates that the improved model enables efficient and reliable fall detection, which has significant practical implications for ensuring the safety of older adults and promoting healthy aging.

## 1 Introduction

Falls represent a serious public health issue worldwide, posing a significant threat especially to the older adult(s) population and being one of the main causes of accidental injuries, functional impairments, and even death (1). According to statistics, approximately 172 million people are injured by falls globally each year, with about 684,000 fatalities (2). As people age, factors such as weakened muscle strength and reduced balance further increase the risk of falls. Falls not only cause physical injuries like fractures and brain damage but also lead to psychological problems such as “fall fear,” which in turn restricts daily activities and affects quality of life and social participation (3). Timely detection of fall incidents and issuance of alerts can save precious time for rescue and significantly reduce the risk of injury. Therefore, the development of real-time and accurate fall detection systems holds significant application value and social significance (4).

Traditional fall detection methods mainly rely on wearable sensors such as accelerometers and gyroscopes, as well as environmental sensors like pressure pads and infrared sensors. Although these approaches can achieve a certain level of fall detection functionality, their long-term application in core older adult(s) care scenarios—such as nursing homes, community-based care centers, outdoor activity areas for the older adult(s), and private residences of older adults living alone—still faces numerous unavoidable limitations. These include insufficient user compliance, discomfort associated with wearing devices, susceptibility to environmental interference, and high deployment and maintenance costs (5, 6). In recent years, computer vision-based fall detection methods for the older adult(s) have effectively addressed the fundamental shortcomings of traditional wearable solutions. These approaches offer the potential advantages of being non-contact, rich in perceptual information, and easy to deploy. By enabling continuous and intuitive analysis of fall actions and postures through techniques such as object detection, pose estimation, or behavior recognition, they operate without disrupting the daily lives of users and eliminate the need for older adults to actively cooperate in wearing devices, thereby reducing the costs associated with large-scale deployment (7, 8).

However, existing visual detection algorithms still generally suffer from high model complexity, sensitivity to lighting and environmental changes, and insufficient generalization ability in real complex scenarios, which restricts their reliable application in actual older adult(s) care monitoring. Among numerous visual object detection models, YOLOv8n, as the latest lightweight model in the YOLO series, holds an important position in the field of real-time object detection. Leveraging its efficient network architecture and flexible modular design, this model has become a foundational framework for numerous vision tasks and is widely applied in resource-constrained environments such as nursing homes. However, in practical deployment and complex scenarios of specific tasks, YOLOv8n still faces issues such as relatively high model complexity, limited generalization ability in different lighting and complex environments, and difficulty in capturing effective discriminative features, which restrict its further application in scenarios requiring high reliability and low resource consumption.

The above limitations ultimately stem from the insufficiency of the model’s architecture in feature extraction, multi-scale information fusion, and the ability to focus on key information. To address these challenges, this study focuses on typical application scenarios such as nursing homes and community activity centers. A multi-pose fall detection database is constructed, encompassing various lighting conditions and diverse real-life scenarios involving older adult(s) individuals. Based on the YOLOv8n model, targeted improvements are implemented to enhance its capability in extracting and discriminating fall-related features. This enables real-time and accurate detection of falls among the older adult(s) under complex lighting conditions in real-world settings.

## 2 Materials and methods

### 2.1 Construction of the dataset

**Figure 1:**
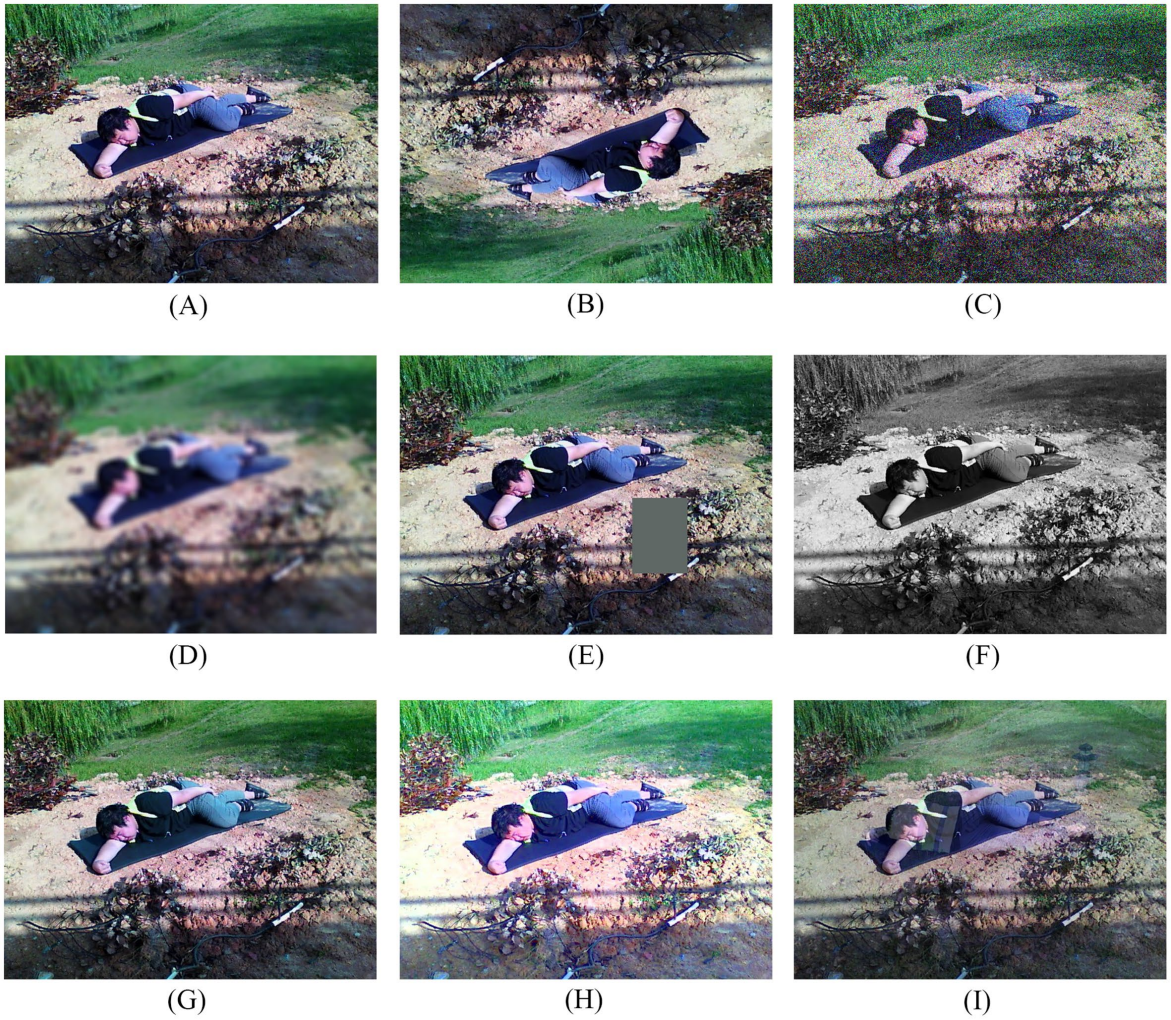
Illustration of data augmentation strategies. **(A)** Original image; **(B)** random flipping; **(C)** noise injection; **(D)** Gaussian blur; **(E)** random erasing; **(F) g**rayscale conversion; **(G) c**olor shifting; **(H)** brightness enhancement; **(I)** mixup.

To address the issue of insufficient data samples in various scenarios and under complex lighting conditions, this study utilized a high-definition RGB camera (1080p, 15 FPS) to collect 1,442 images of human falls under various typical complex scenarios, including residences, corridors, parks, lawns, and squares, under different lighting intensities (such as direct strong light, dim light, backlighting, and normal indoor lighting). A total of 1,442 original images were collected. Data augmentation techniques including random flipping, noise injection, Gaussian blur, random erasing, grayscale conversion, color shifting, brightness enhancement, and Mixup were subsequently applied to enrich the dataset. This process yielded 7,921 fall image samples and 3,615 non-fall image samples, resulting in a total of 11,536 images. The dataset comprises 2,144 images of residences, 2,296 of corridors, 2,344 of parks, 2,568 of lawns, and 2,184 of squares. In terms of lighting distribution, there are 1,902 images captured under direct strong light, 3,318 under dim light, 1,758 under backlight, and 1,674 under indoor ambient light. The effect of data augmentation is illustrated in Figure 1.

**Figure 2:**
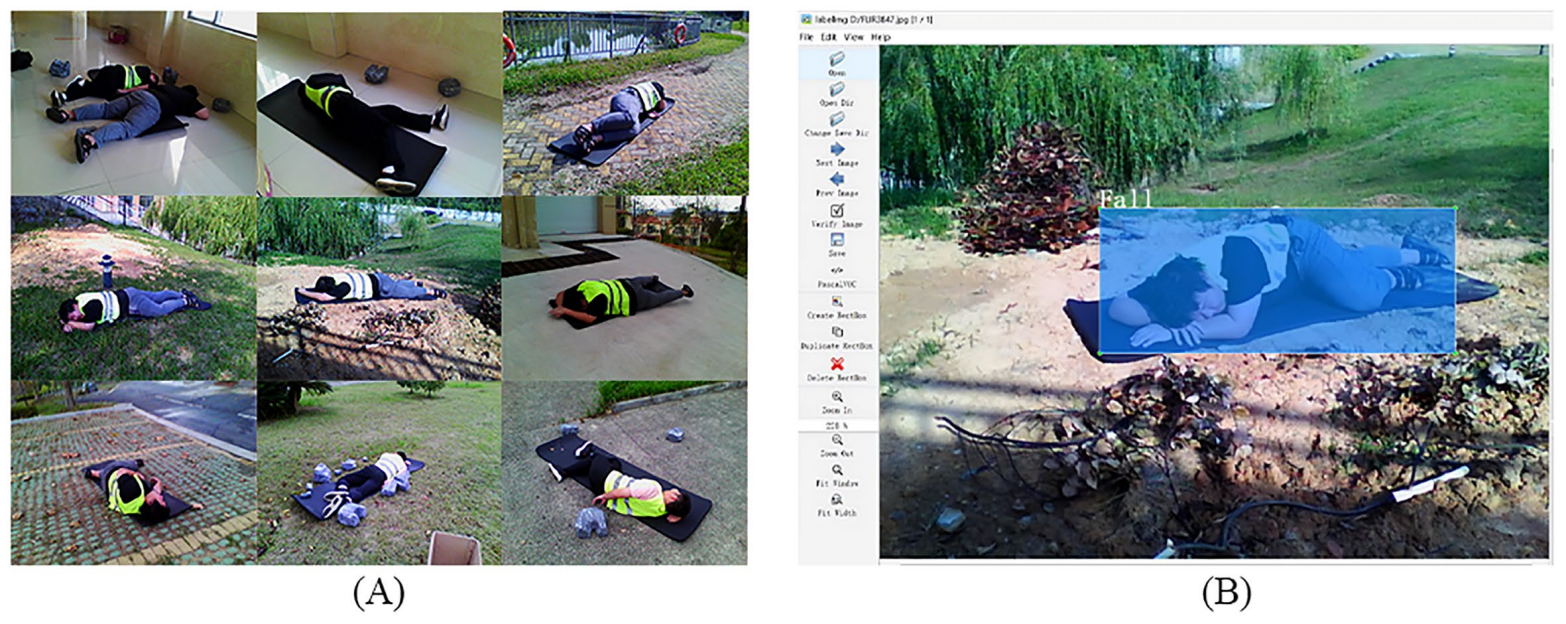
The construction process of the fall detection dataset, including data collection and labeling steps. **(A)** Fall-related data: a set of 9 images (arranged in 3 rows and 3 columns) showing representative fall scenario samples, which were collected under diverse environmental conditions (e.g., outdoor, indoor) and light intensities (e.g., strong illumination, weak illumination); **(B)** Data labeling: example of labeling the fall target area in the collected image (the blue box indicates the region of interest, and the “fall” label corresponds to the detected action category).

Finally, the LabelImg tool was employed to manually annotate each image and perform rigorous reviews, resulting in the construction of a high-quality fall event database encompassing multiple scenarios and complex lighting conditions. The database was subsequently partitioned into training, validation, and test sets with a ratio of 8:1:1, as detailed in Figure 2.

### 2.2 Experimental design

This study is based on YOLOv8n and systematically introduces multiple advanced lightweight and attention mechanism modules, including RepVGG, SPPF_LSKA, SimSPPF, Shuffle Attention, SEAttention, C2f_ScConv, and C2f_PKI. Through the design of single-module, dual-module, and triple-module fusion improvement strategies, systematic ablation experiments are conducted to explore the coupling relationship among different improvement strategies and their gain effects on the overall performance of the model.

#### 2.2.1 YOLOv8 network structure

**Figure 3:**
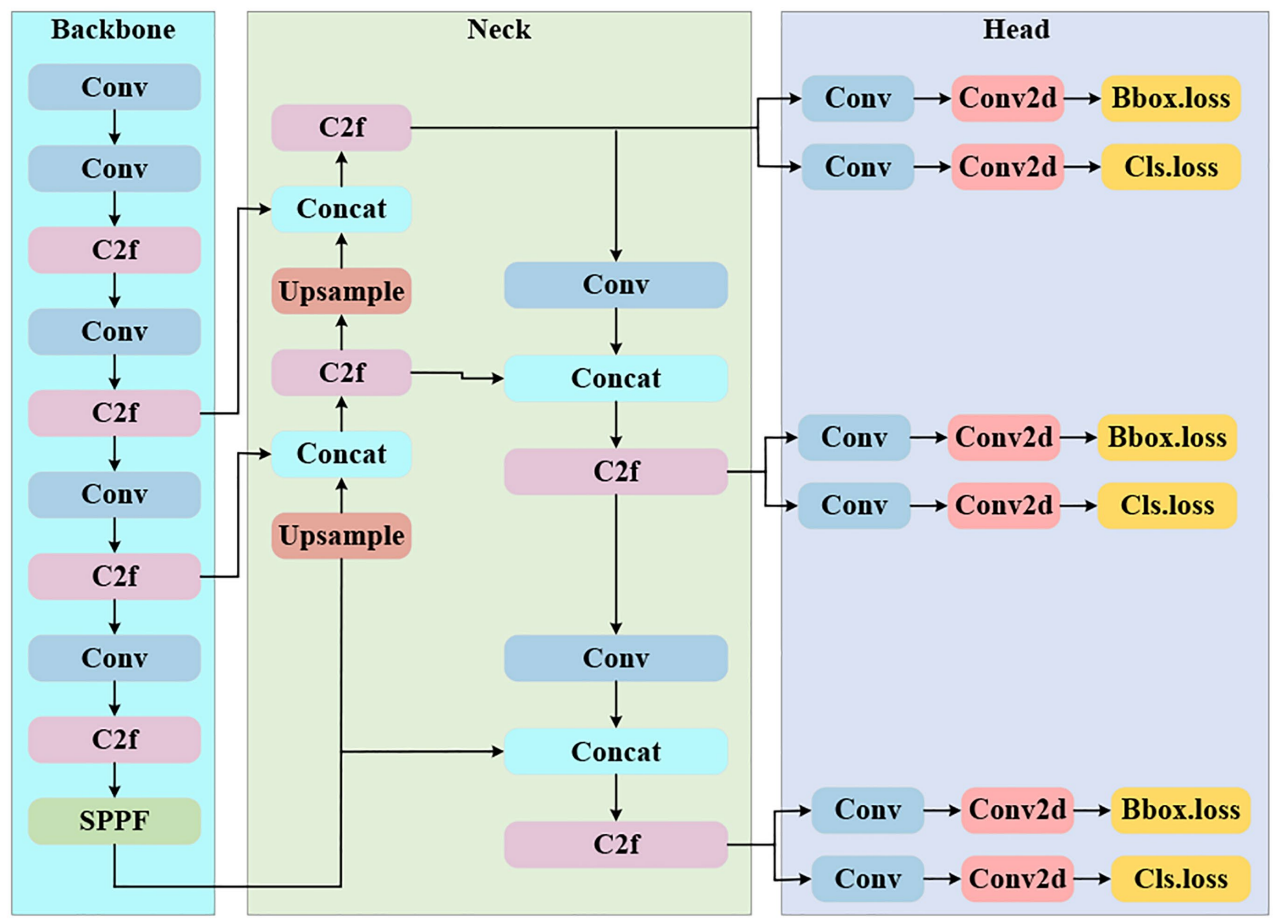
The network architecture of YOLOv8, which consists of three functional modules: backbone, neck, and head. (1) Backbone: extracts multi-scale features through convolutional layers (Conv), C2f modules (feature fusion blocks), and an SPPF module (spatial pyramid pooling); (2) Neck: performs feature fusion and scale adjustment via up sample (up-sampling) and Concat (feature concatenation) operations, combined with C2f and Conv modules; (3) Head: generates detection results by outputting bounding box loss (Bbox_loss) and classification loss (Cls_loss) through Conv and Conv2d layers, corresponding to different feature scales.

YOLOv8 (9, 10) is the latest iteration of the YOLO series of object detection algorithms, and its overall framework consists of three parts: Backbone, Neck, and Head. The Backbone employs a series of convolutional (Conv) and C2f modules for hierarchical feature extraction and introduces the SPPF module at the end to perform spatial pyramid pooling, thereby enhancing the model’s ability to perceive multi-scale objects. The feature fusion network (Neck) adopts a bidirectional feature pyramid structure combining FPN and PAN, utilizing upsampling and concatenation operations to supplement and strengthen low-level detail features from the bottom up, while transmitting and refining high-level semantic features from the top down, effectively addressing the information fusion among multi-level feature maps. The detection head (Head) adopts a Decoupled-Head structure, which first further enhances the fused features through a C2f module, and then feeds them into the classification branch and regression branch, respectively. Each branch consists of convolutional layers (Conv) and Conv2d layers, which compute the classification loss (Cls.loss) and bounding box loss (Bbox.loss) in parallel, ultimately achieving efficient and accurate object detection. The overall network structure is shown in Figure 3. YOLOv8 is available in five variants: YOLOv8n, YOLOv8s, YOLOv8m, YOLOv8l, and YOLOv8x, among which YOLOv8n is the smallest model with the fastest detection speed. Based on this, YOLOv8n is adopted as the baseline model for this study.

#### 2.2.2 Reparameterization Visual Geometry Group (RepVGG) module

The Reparameterization Visual Geometry Group (RepVGG) module (11, 12) is a “training-inference decoupled” convolutional neural network architecture. It draws on the idea of ResNet and adopts a multi-branch topology in the training phase, which includes a 3 × 3 convolutional layer as the backbone, a 1 × 1 convolutional layer with a residual connection, and an identity mapping directly connected to the output, to enhance gradient flow and feature expression capabilities. In the inference phase, the multi-branch structure is equivalently transformed into a single 3 × 3 convolutional layer through parameter fusion, thereby maintaining strong representational power while achieving efficient and concise inference performance similar to that of VGG.

This study is based on the idea of Structural Re-parameterization and introduces RepVGG blocks in the Backbone section to replace some of the standard convolution operations in the original C2f module, which can effectively enhance the feature extraction capability of the model and enable deployment on edge devices with limited computing resources (13).

#### 2.2.3 Squeeze-and-Excitation Attention (SEAttention) module

The Squeeze-and-Excitation Attention (SEAttention) module (14, 15) is an efficient channel attention mechanism derived from the Squeeze-and-Excitation Network. By explicitly modeling the interdependencies among convolutional feature channels, it adaptively calibrates the channel feature responses, enabling the network to focus more on channels with greater information content. This mechanism first performs a Squeeze operation on the input feature map through global average pooling to compress spatial information and generate a channel description vector. Then, in the Excitation operation, a bottleneck gating mechanism is used to learn the nonlinear dependencies among channels and generate channel weights. Finally, the Scale operation multiplies the weights with the original features channel-wise to achieve channel feature recalibration and enhancement.

In this experiment, the SEAttention mechanism is introduced into the multi-scale feature fusion layer of Neck, which can adaptively enhance the important channel features and suppress redundant information, thereby improving the model’s perception ability for key features and detection accuracy (16).

#### 2.2.4 Shuffle Attention (SA) module

The Shuffle Attention (SA) module (17, 18) is a lightweight attention mechanism that combines channel and spatial attention. By employing Grouping and Channel Shuffle strategies, it achieves cross-dimensional feature interaction and calibration at a low computational cost. This module divides the input features into multiple groups along the channel dimension and performs channel attention and spatial attention in parallel within each group. It extracts channel statistical information through global pooling and spatial salient regions through group normalization, and then calibrates the features. The processed sub-features are then concatenated, and a channel shuffle operation promotes inter-group information fusion, achieving global feature interaction and enhancement, thereby effectively improving the model’s representation capability.

In this experiment, the Shuffle Attention mechanism is introduced into the neck network of YOLOv8n on the multi-scale feature fusion path. It can simultaneously capture the inter-channel dependencies and spatial feature relationships through channel re-grouping and parallel processing, thereby enhancing the network’s feature extraction capabilities for small and occluded objects (19).

#### 2.2.5 Spatial Pyramid Pooling with Fast Large Separable Kernel Attention (SPPF_LSKA) module

The Large Separable Kernel Attention (LSKA) module is an efficient spatial attention mechanism that decomposes the traditional large convolutional kernel into three steps: depthwise convolution, dilated depthwise convolution, and 1 × 1 convolution. This approach enables the network to obtain a global receptive field while significantly reducing computational costs, allowing it to focus on key spatial regions. The Spatial Pyramid Pooling with Fast Large Separable Kernel Attention (SPPF_LSKA) module (20) further enhances this by integrating the SPPF structure before LSKA. It fuses context information from different receptive fields through multi-scale max pooling and feature concatenation, and then feeds the rich multi-scale features into LSKA for attention weighting. This process not only suppresses noises but also more precisely enhances key features.

This study employs SPPF_LSKA to replace the original SPPF module, enabling the model’s neck network to simultaneously perform global semantic association calculations during the process of multi-scale feature fusion. The aim is to more effectively integrate feature information at different levels and ultimately enhance the model’s accuracy and robustness in recognizing targets in complex scenarios (21).

#### 2.2.6 Simplified Spatial Pyramid Pooling-Fast (SimSPPF) module

**Figure 4:**
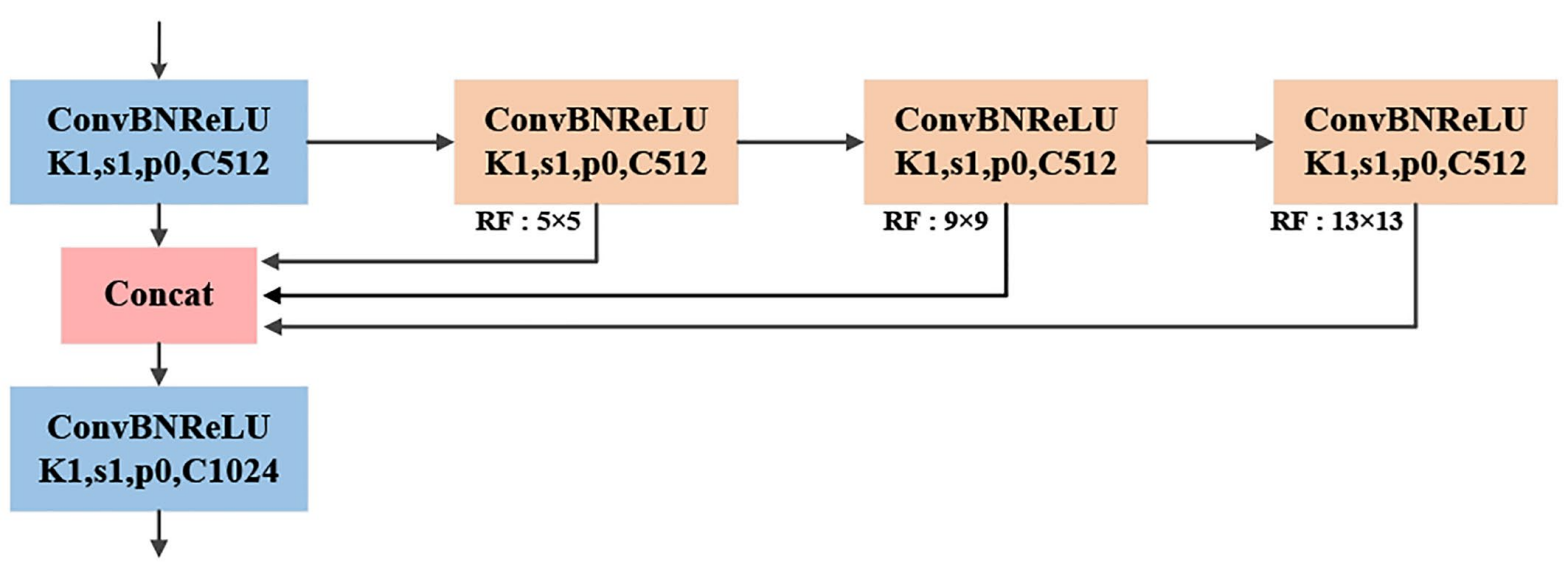
The architecture of the SimSPPF module, a feature extraction component with multi-receptive field fusion. The module consists of four parallel ConvBNReLU layers (all with kernel size *K* = 1, stride *s* = 1, padding *p* = 0, output channel *C* = 512), which correspond to receptive fields (RF) of 5 × 5, 9 × 9, and 13 × 13 (the first layer serves as the base feature branch). The outputs of these four branches are concatenated via the Concat operation, and finally processed by a ConvBNReLU layer (*C* = 1,024) to generate the fused feature.

The Simplified Spatial Pyramid Pooling-Fast (SimSPPF) module (22, 23) is a computational path optimization of the original SPPF module in YOLOv8. It retains the core advantage of multi-scale receptive fields while achieving a faster inference speed by simplifying the feature fusion path, making it a lightweight improvement solution that balances performance and efficiency. The SimSPPF inherits the core structure of the SPPF, which consists of concatenated max pooling layers of different sizes, including 5 × 5, 9 × 9, and 13 × 13, as shown in Figure 4.

The original SPPF feeds the input feature map successively into pooling layers of different sizes to obtain outputs at multiple scales. Then, all the outputs are concatenated along the channel dimension, and finally fused through a convolutional layer. Its path can be simplified as Equation (1):


$$\text{Output}=\text{Conv}(\text{Concat}(\begin{array}{l} X,{\text{MaxPool}}_{3}(X),{\text{MaxPool}}_{2}\\ \left(X),{\text{MaxPool}}_{1}(X\right) \end{array}))$$ (1)

SimSPPF adopts a cumulatively concatenated strategy. The output of the current pooling layer directly serves as the input for the next pooling layer with a larger kernel size, and ultimately only the initial input and the output of the final pooling layer are concatenated. The formula can be expressed as Equation (2):


$$\text{Output}=\text{Conv}(\text{Concat}(\begin{array}{l} X,{\text{MaxPool}}_{3}\\ \left({\text{MaxPool}}_{2}({\text{MaxPool}}_{1}(X))\right) \end{array}))$$ (2)

This study replaces SPPF with SimSPPF, which maintains multi-scale feature fusion performance while significantly reducing the computational complexity of the model, there by enhancing the real-time performance and deployment efficiency of the detection system (24, 25).

#### 2.2.7 C2f module with spatial and channel reconstruction convolution (C2f_ScConv)

The C2f module with Spatial and Channel Reconstruction Convolution (C2f_ScConv) (26) is based on the multi-branch gradient flow design of the original Convolution to Feature (C2f) structure, replacing the standard convolution with the Spatial and Channel Reconstruction Convolution (ScConv) unit. The ScConv unit consists of two key components: the Spatial Reconstruction Unit and the Channel Reconstruction Unit. The SRU enhances the feature response of important spatial regions through the generation of separation masks and feature selection; the CRU improves the efficiency of information integration among feature channels through channel splitting and cross-channel fusion. The combination of the two enables the module to maintain rich gradient flow while effectively reducing redundant computations to enhance the model’s feature representation ability (27).

In this experiment, the C2f_ScConv module was introduced into the backbone network to replace the C2f module, significantly reducing the number of parameters and computational load while maintaining the model’s accuracy, effectively enhancing the feature extraction efficiency and lightweight level of the convolutional network (28).

#### 2.2.8 C2f module with partial kernel interaction (C2f_PKI)

**Figure 5:**
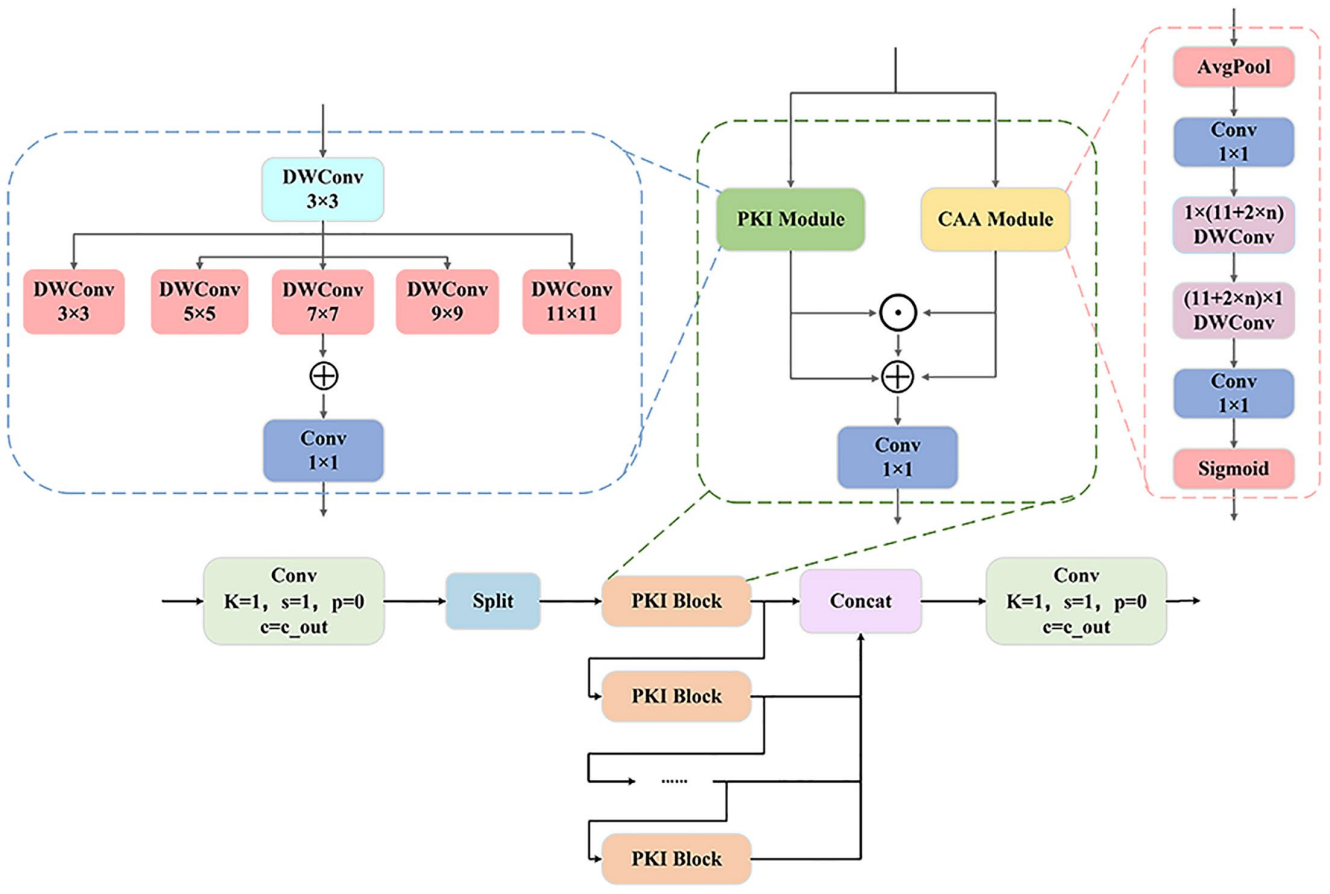
The architecture of the C2f_PKI module, a feature enhancement component with multi-branch and attention mechanisms.

The C2f module with Partial Kernel Interaction (C2f_PKI) (29) is a deep optimization of the C2f structure in YOLOv8, where the Bottleneck sub-module in the original C2f module is replaced with the PKI Block. The PKI Block is a lightweight module designed to address the background noise and feature sparsity issues introduced by large convolution kernels. It is composed of a PKI module and a CAA module in cascade. The PKI module adopts a non-dilated Inception structure, extracting multi-scale local context features through multiple parallel depthwise convolutions and fusing them via a 1 × 1 convolution to enhance the ability to capture textures and details. The CAA Module first aggregates spatial information through average pooling and a 1 × 1 convolution, then establishes long-range cross-pixel dependencies using a pair of asymmetric depthwise separable convolutions with dimensions of 1 × (11 + 2n) and (11 + 2n) × 1, and finally outputs channel attention weights to enhance important features and suppress noise (30). Working together, the two modules significantly enhance the model’s multi-scale feature representation ability and robustness in detecting small objects in complex scenes while maintaining efficiency, as shown in Figure 5.

This paper integrates the C2f_PKI module into the YOLOv8n backbone network, replacing the first standard C2f module, which has a quantifiable positive impact. It enables the model to incorporate more refined feature modulation capabilities at the key feature abstraction level with little change in computational burden (31).

### 2.3 Module improvement solutions

To explore the performance improvement effects of each modified module and select the best model improvement strategy, this study conducted systematic experiments involving single-module, dual-module, and triple-module integrated improvements based on the aforementioned lightweight and attention mechanism modules. The specific combinations of enhancement strategies are detailed in Table 1.

**Table 1:** Model improvement solutions.

Model solution	Solution	RepVGG	Shuffle Attention	SE Attention	SPPF_LASK	SimSPPF	C2f_ScConv	C2f_PKI
Single-module integrated improvements	1	✓						
	2		✓					
	3			✓				
	4				✓			
	5					✓		
	6						✓	
	7							✓
Dual-module integrated improvements	8	✓	✓					
	9		✓		✓			
	10	✓			✓			
	11				✓		✓	
	12		✓				✓	
	13			✓				✓
	14					✓		✓
	15			✓		✓		
Triple-module integrated improvements	16	✓	✓		✓			
	17		✓			✓		✓
	18			✓	✓			✓
	19			✓		✓	✓	

## 3 Results

### 3.1 Experimental environment

This experiment utilized a computer platform equipped with a 13th generation Intel Core i5-13500HX processor and an NVIDIA GeForce RTX 4060 Laptop GPU (8GB VRAM), along with 16GB of memory, to provide computational power for deep learning model training. In terms of software, Windows 11 was selected as the operating system, and a development environment was built based on Python 3.9.19. The PyTorch 2.8.0 deep learning framework and CUDA 12.4 toolkit were employed to effectively invoke and accelerate GPU computing resources in parallel. Detailed information is shown in Table 2.

**Table 2:** Configuration and training environment.

Software/hardware	Version
Operating system	Windows 11
CPU	13th Gen Intel(R) Core(TM) i5-13500HX 2.50 GHz
GPU	NVIDIA GeForce RTX 4060 Laptop GPU
VRAM	8GB
RAM	16GB
Programming Language Version	Python-3.9
GPU Parallel Computing Platform	CUDA 12.4
Deep Learning Framework	torch-2.8.0 + cu126
IDE	Pycharm

The basic information of the model training parameters is shown in Table 3.

**Table 3:** Training parameters.

Training parameters	Value
Epochs	100
Image Size	640 × 640
Batch Size	16
Optimizer	SGD
workers	0
device	CPU

### 3.2 Evaluation indicators

In this study, the detection results are evaluated using the mean average precision mAP@50, Precision, FPS, and Recall metrics. Considering the one-sidedness of evaluating the model solely by Precision and Recall, which may cause certain deviations in the assessment of detection performance, this paper will focus on the performance of each model in terms of the mAP@50 value. This metric reflects the overall balance performance achieved by the model between precision and recall, and can more accurately and comprehensively evaluate the comprehensive performance of the model.

SimSPPF adopts a cumulatively concatenated strategy. The output of the current pooling layer directly serves as the input for the next pooling layer with a larger kernel size, and ultimately only the initial input and the output of the final pooling layer are concatenated. The formula can be expressed as Equation (2):

Precision refers to the proportion of actual fall events among the samples predicted as falls by the classification model. The calculation formula is shown as Equation (3):


$$\text{Precision}=\frac{\mathrm{TP}}{\mathrm{TP}+\mathrm{FP}}$$ (3)

Among these, TP represents True Positives, TN represents True Negatives, FP represents False Positives, and FN represents False Negatives.

SimSPPF adopts a cumulatively concatenated strategy. The output of the current pooling layer directly serves as the input for the next pooling layer with a larger kernel size, and ultimately only the initial input and the output of the final pooling layer are concatenated. The formula can be expressed as Equation (2):

Recall measures the proportion of actual fall events that are correctly identified by the model out of the total number of actual fall events, reflecting its ability to avoid missed detections. The calculation formula can be expressed as Equation (4):


$$\text{Recall}=\frac{\mathrm{TP}}{\mathrm{TP}+\mathrm{FN}}$$ (4)

SimSPPF adopts a cumulatively concatenated strategy. The output of the current pooling layer directly serves as the input for the next pooling layer with a larger kernel size, and ultimately only the initial input and the output of the final pooling layer are concatenated. The formula can be expressed as Equation (2):

Mean Average Precision is a comprehensive metric that assesses the model’s performance in distinguishing between the “fall” and “non-fall” categories across various recall thresholds. As the core evaluation metric, it is the primary focus of this paper. It can be calculated by Equation (5):


$$\mathrm{mAP}=\frac{1}{n\;}\sum_{i=1}^{n}{\mathrm{AP}}_{i}$$ (5)

SimSPPF adopts a cumulatively concatenated strategy. The output of the current pooling layer directly serves as the input for the next pooling layer with a larger kernel size, and ultimately only the initial input and the output of the final pooling layer are concatenated. The formula can be expressed as Equation (2):

FPS measures the number of image frames the model can process per second in an actual operating environment, providing a more intuitive reflection of the model’s operational efficiency and real-time responsiveness. This can be formulated as Equation (6):


$$\mathrm{FPS}=\frac{1}{T\;}$$ (6)

T represents the time consumed to process one frame of an image.

In addition, Parameters and GFLOPs are also adopted as evaluation metrics in this study, aiming to comprehensively assess the real-time deployment potential of the improved model from the two dimensions of model storage cost and computational efficiency per frame, respectively.

To verify the advantages and differences of various improvement strategies of YOLOv8n in fall detection, we conducted a comparative analysis of the performance metrics based on single-module, dual-module, and triple-module fusion improvements on the fall dataset. The experimental results are shown in Tables 4–6.

**Table 4:** Performance comparison of YOLOv8n baseline and its variants with single-module improvement strategies.

Model design	Precision	Recall	mAP@0.5	Parameters/M	GFLOPs
YOLOv8n (Baseline)	91.3%	78.9%	89.7%	3.0	8.1
YOLOv8n-RepVGG	87.3%	85.7%	91.7%	3.0	8.1
YOLOv8n-ShuffleAttention	88.3%	76.9%	90.3%	3.0	8.1
YOLOv8n-SEAttention	87.8%	81.0%	88.9%	3.0	8.1
YOLOv8n-SPPF_LSKA	83.5%	89.8%	91.3%	3.3	8.3
YOLOv8n-SimSPPF	83.2%	85.0%	90.3%	3.0	8.0
YOLOv8n-C2f_ScConv	79.7%	74.7%	83.5%	2.8	7.5
YOLOv8n-C2f_PKI	87.7%	81.0%	88.3%	3.0	8.3

**Table 5:** Performance comparison of YOLOv8n baseline and its variants with dual-module integration strategies.

Model design	Precision	Recall	mAP@0.5	Parameters (M)	GFLOPs
YOLOv8n (Baseline)	91.3%	78.9%	89.7%	3.0	8.1
YOLOv8n-RepVGG+ShuffleAttention	84.4%	80.9%	87.9%	3.0	8.1
YOLOv8n-ShuffleAttention+SPPF_LSKA	85.5%	76.4%	88.0%	3.3	8.3
YOLOv8n-RepVGG+SPPF_LSKA	86.2%	78.9%	87.9%	3.3	8.3
YOLOv8n-C2f_ScConv+SPPF_LSKA	72.1%	78.9%	83.6%	3.1	7.7
YOLOv8n-C2f_ScConv+ShuffleAttention	78.9%	71.2%	81.5%	2.8	7.5
YOLOv8n-C2f_PKI + SEAttention	79.5%	83.0%	87.6%	3.0	8.3
YOLOv8n-C2f_PKI + SimSPPF	86.6%	83.8%	91.8%	3.0	8.3
YOLOv8n-SimSPPF+SEAttention	80.8%	85.0%	90.1%	3.0	8.1

**Table 6:** Performance comparison of YOLOv8n baseline and its variants with tri-module integration strategies.

Model design	Precision	Recall	mAP@0.5	Parameters (M)	GFLOPs
YOLOv8n (Baseline)	91.3%	78.9%	89.7%	3.0	8.1
YOLOv8n-RepVGG+SPPF_LSKA+ShuffleAttention	85.1%	77.8%	86.9%	3.3	8.3
YOLOv8n-C2f_PKI + SimSPPF+ ShuffleAttention	86.3%	78.2%	88.8%	3.0	8.3
YOLOv8n-C2f_PKI + SPPF_LSKA+SEAttention	88.8%	80.9%	88.3%	3.3	8.5
YOLOv8n-C2f_ScConv+SimSPPF+SEAttention	84.9%	74.8%	85.6%	2.8	7.5

### 3.3 Single-module improvement results

In the single-module improvement strategy, each improved module had a differentiated impact on the baseline model YOLOv8n, showing different performance tendencies. Experiments demonstrated that YOLOv8n-RepVGG achieved significant improvements in recall and mAP@0.5 while maintaining the same number of parameters and GFLOPs as YOLOv8n. On the validation set, its Recall reached 85.7%, a 6.8% increase over the baseline model, and its mAP@0.5 reached 91.7%, a 2.1% increase over the baseline model, indicating that its structural reparameterization design effectively enhanced the model’s feature fusion and generalization capabilities. YOLOv8n-SPPF_LSKA performed best in terms of recall, reaching 89.8%, and its mAP@0.5 was also higher than that of YOLOv8n, suggesting that this module significantly enhanced the model’s target coverage ability, especially suitable for scenarios with high missed detection rates, although its parameter count and computational cost slightly increased. In contrast, YOLOv8n-C2f_ScConv achieved a significant reduction in parameters and GFLOPs, with a 6.7% reduction in parameters and a 7.4% reduction in computational cost compared to YOLOv8n, making the model more lightweight. However, its three key detection metrics, Precision, Recall, and mAP@0.5, all showed a significant decline, indicating that while compressing the model, this module caused a certain loss in feature representation ability. Nevertheless, most attention mechanisms and lightweight modules, such as ShuffleAttention, SEAttention, SimSPPF, and C2f_PKI, brought limited accuracy gains, and some even led to a decline in key metrics.

**Figure 6:**
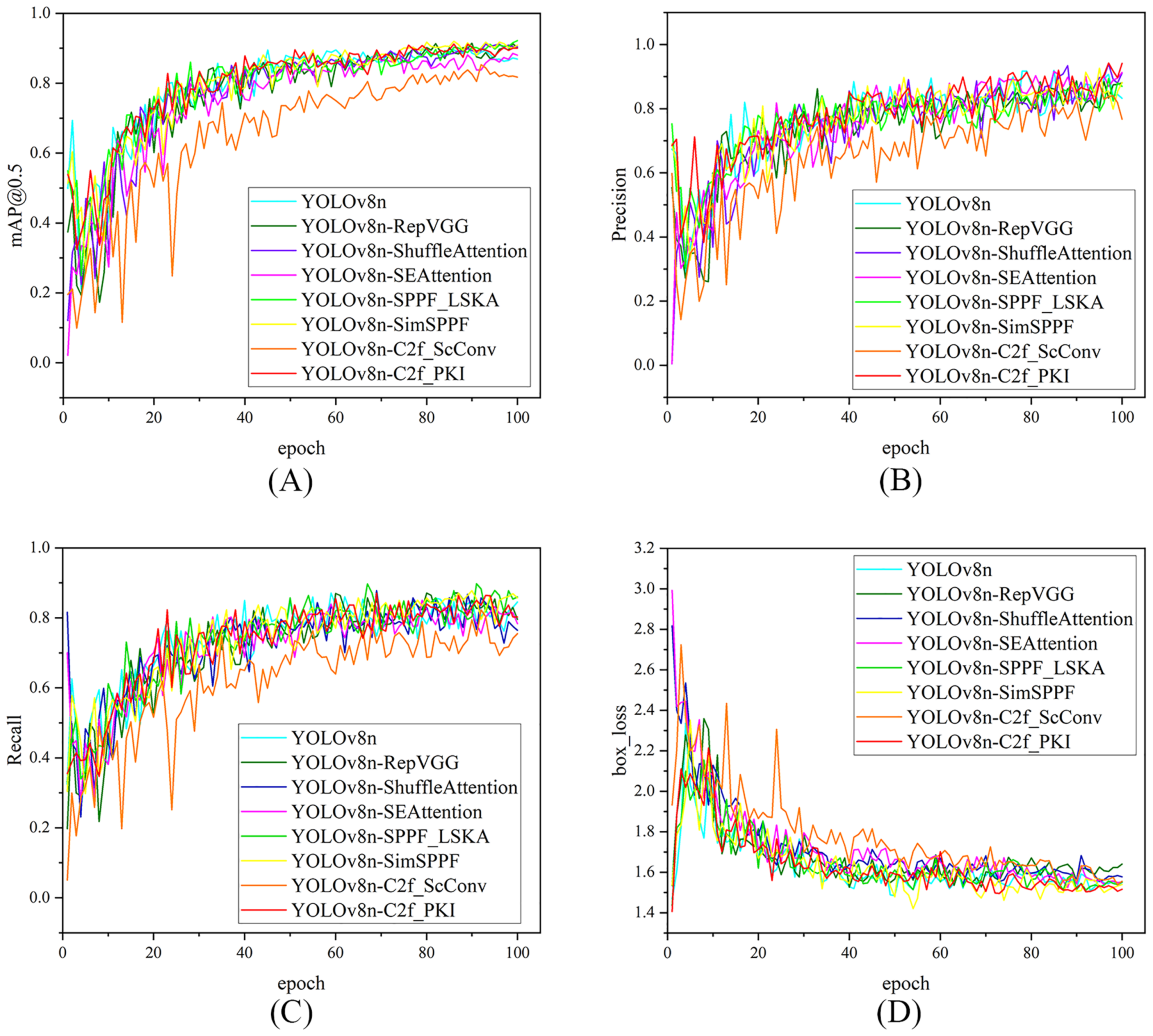
Comparison of model performance metrics (across 100 training epochs) on the validation set, for different single-module improvement strategies based on YOLOv8n. The 4 subplots (arranged in 2 × 2 grid) show: **(A)** mAP@50 curve (mean average precision at IOU = 0.5), **(B)** Precision curve (detection accuracy), **(C)** Recall curve (detection coverage), and **(D)** Bbox loss curve (bounding box regression loss). Each curve corresponds to a variant (e.g., YOLOv8n-SimSPPF, YOLOv8n-C2f_PKI), demonstrating the performance changes of different module optimization strategies during training.

Comprehensive analysis indicates that YOLOv8n-RepVGG is the improved model with the best overall performance among single-module models. It effectively enhances the feature representation ability through structural reparameterization technology, thereby achieving simultaneous improvements in recall rate and mAP@50 while maintaining the same number of parameters and computational cost. This result validates that introducing efficient structural optimization in lightweight architectures is an effective way to enhance the comprehensive performance of full detection models. However, the decline in precision of this model also highlights the common challenge of the precision-recall trade-off in object detection. The detection performance of each model on the validation set is shown in Figure 6.

### 3.4 Dual-module integration improvement results

**Figure 7:**
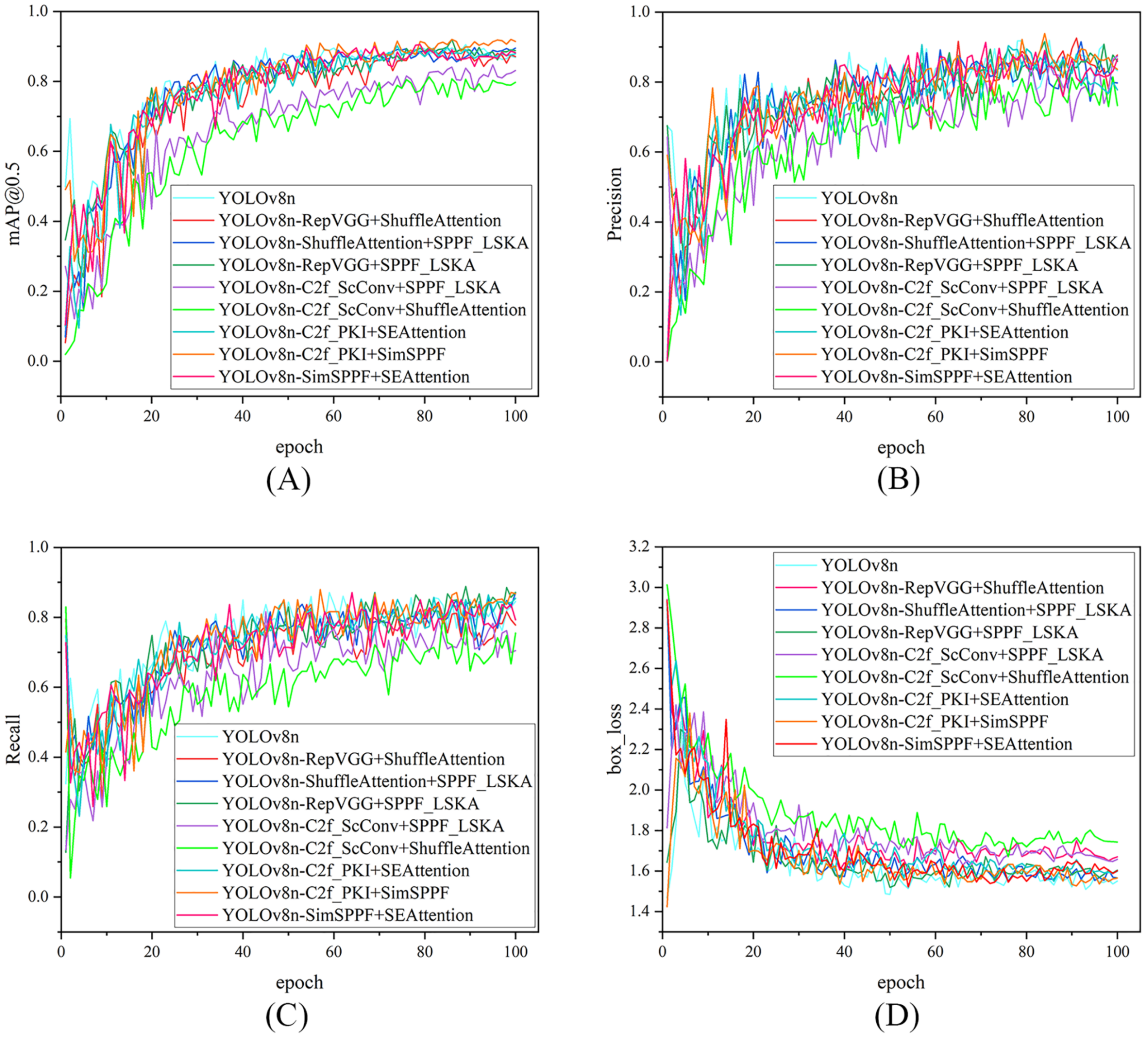
Comparison of model performance metrics (over 100 training epochs) on the validation set, for different dual-module integration strategies based on YOLOv8n. The 4 subplots (arranged in a 2 × 2 grid) are as follows: **(A) S**hows the mAP@50 curve (mean average precision at IOU = 0.5); **(B)** shows the precision curve (detection accuracy); **(C)** shows the recall curve (detection coverage); **(D)** shows the Bbox loss curve (bounding box regression loss). Each curve corresponds to a dual-module optimized variant (e.g., YOLOv8n-C2f_PKI + SimSPPF, YOLOv8n-SimSPPF+SEAttention), demonstrating the training process performance changes of different combined module improvement strategies.

Experiments on various dual-module combination improvements to YOLOv8n show that most dual-module fast fusion improvement strategies outperform the 78.9% recall rate of the YOLOv8n model. Notably, the dual-module fast combination of SimSPPF and SEAttention achieves a recall rate of 85.0%, indicating that such structures can enhance the model’s coverage of targets. However, the precision of almost all improved models is lower than the 91.3% of the baseline model, with some combinations dropping significantly to 72.1%, such as the fusion strategy of C2f_ScConv and SPPF_LSKA, suggesting that while module fusion enhances recall, it often leads to an increase in false detections. Considering the mean average precision at 0.5 IoU (mAP@0.5) as an indicator of overall detection performance, the fusion strategy of C2f_PKI and SimSPPF performs best, reaching 91.7%, surpassing the 89.7% of the baseline model, and simultaneously increasing the recall rate to 83.8%, demonstrating a good synergy in feature extraction and multi-scale fusion. In contrast, combinations involving C2f_ScConv, although significantly reducing the number of parameters and computational cost, show a marked decline in mAP@0.5 and precision, indicating that lightweight design requires a careful balance between efficiency and performance. From the perspective of structural combination effects, the introduction of attention mechanisms and reparameterization does not consistently lead to performance improvements; some combinations, such as RepVGG and ShuffleAttention, suffer losses in both precision and mAP@0.5. This suggests that the interaction between modules is complex, and not all combinations can achieve positive gains, necessitating further ablation studies to determine the optimal embedding positions and collaborative mechanisms of each module. The detection performance of each model on the validation set is detailed in Figure 7.

### 3.5 Triple-module integration improvement results

Table 6 presents a performance comparison between the YOLOv8n baseline model and four three-module fusion improvement models. Overall, the three-module fusion did not comprehensively outperform the baseline model in terms of overall performance. The mAP@0.5 of all the improvement schemes was lower than the 89.7% of the baseline, and the precision generally declined, further confirming the common performance offset effect in multi-module integration. Among them, the three-module fusion strategy of C2f_PKI, SPPF_LSKA, and SEAttention performed best in terms of precision and recall rate, with a precision of 88.8% and a recall rate of 80.9%, but the corresponding number of parameters and computational cost were also the highest, reflecting a certain performance-resource trade-off. In contrast, the fusion strategy of C2f_ScConv, SimSPPF, and SEAttention significantly reduced the model complexity, with the number of parameters dropping to 2.8 M and GFLOPs to 7.5, but it led to a simultaneous decline in other detection metrics, indicating that lightweight design in multi-module fusion may exacerbate the loss of feature expression ability. Notably, the fusion strategy of C2f_PKI, SimSPPF, and ShuffleAttention maintained a similar number of parameters and computational cost to the baseline while achieving a comprehensive detection performance mAP@0.5 close to the YOLOv8n baseline model, suggesting that this combination achieved a certain balance among feature extraction, multi-scale fusion, and attention mechanisms, but its precision still lagged behind the baseline.

**Figure 8:**
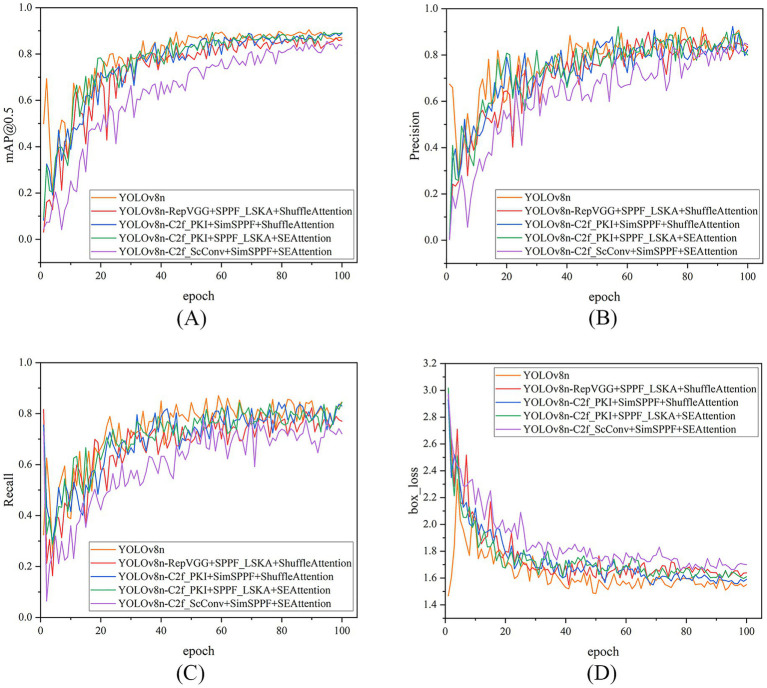
Comparison of model performance metrics (over 100 training epochs) on the validation set, for different tri-module integration strategies based on YOLOv8n. The 4 subplots (arranged in a 2 × 2 grid) are as follows: **(A)** Shows the mAP@50 curve (mean average precision at IoU = 0.5); **(B)** shows the precision curve (detection accuracy); **(C)** shows the recall curve (detection coverage); **(D)** shows the Bbox loss curve (bounding box regression loss). Each curve corresponds to a tri-module optimized variant (e.g., YOLOv8n-C2f_PKI + SimSPPF+ShuffleAttention, YOLOv8n-C2f_PKI + SPPF_LSKA+SEAttention), demonstrating the training performance trends of different three-module combined improvement strategies.

Furthermore, the experimental results on the validation set for each model show that the Precision, Recall, and mAP@50 values of all models increase with the number of training rounds and eventually converge. However, none of the performance curves of the improved models can surpass that of YOLOv8n. This indicates that, under the experimental setup and evaluation metrics of this study, the specific module combinations attempted did not lead to improvements in Precision, Recall, and mAP@50, and even showed a slight decline. This suggests that the integration and improvement of modules require more meticulous tuning and evaluation, and cannot rely solely on module stacking. Meanwhile, the box_loss steadily decreases throughout the training process and eventually stabilizes, verifying the effectiveness of these improvement strategies in enhancing the model’s localization performance. See Figure 8 for details.

### 3.6 Performance comparison of optimal models on the independent test set

To evaluate the generalization capability of the models, we tested the trained optimal single-module, dual-module, and triple-module fusion models on an independent test set. This test set accounts for 10% of the entire dataset (a total of 1,153 images). The performance metrics of each model are presented in Table 7.

**Table 7:** Performance comparison of optimal models on the independent test set.

Model design	Precision	Recall	mAP@0.5	Parameters (M)	GFLOPs
YOLOv8n (Baseline)	75.9%	75.9%	76.9%	3.0	8.1
YOLOv8n-RepVGG	79.3%	73.5%	76.3%	3.0	8.1
YOLOv8n-C2f_PKI + SimSPPF	81.0%	72.6%	79.6%	3.0	8.3
YOLOv8n-C2f_PKI + SimSPPF+ ShuffleAttention	77.0%	71.7%	76.5%	3.0	8.3

The evaluation results on the independent test set indicate that different improvement strategies have a significant impact on the generalization performance of the models. Among these, the dual-module fusion improvement strategy, YOLOv8n-C2f_PKI + SimSPPF, performs the best, achieving an mAP@0.5 of 79.6%, which is a 2.7% improvement over the baseline model. Its precision increases to 81.0%, while the number of parameters remains unchanged and GFLOPs only marginally increase by 0.2. The model’s recall shows a slight decrease compared to the baseline model. Therefore, a trade-off between precision and recall needs to be considered based on the specific application scenario.

### 3.7 Comparative experiments of representative models

To further validate the superiority of the improved model, comparative experiments were conducted between the enhanced model and classic models including YOLOv5s, YOLOv7t, Faster-RCNN, and MobileNetv3-YOLO, which represent single-stage, two-stage, and lightweight edge paradigms, on the same dataset and under identical training parameters. The results are presented in Table 8.

**Table 8:** Comparative experiments of different typical models.

Model design	Precision	Recall	mAP@0.5	Parameters (M)	GFLOPs	FPS
YOLOv5s	89.6%	85.7%	89.9%	7.0	15.8	19.8
YOLOv7t	89.4%	80.9%	89.3%	6.0	13.2	26.4
Faster-RCNN	82.8%	87.8%	90.7%	2.6	10.7	32.3
MobileNetv3-YOLO	79.8%	78.7%	85.8%	2.5	4.7	39.6
YOLOv8n	91.3%	78.9%	89.7%	3.0	8.1	28.7
YOLOv8n-ours	86.6%	83.8%	91.8%	3.0	8.3	41.6

The experimental results demonstrate that the improved model, YOLOv8n-ours (YOLOv8n-C2f_PKI + SimSPPF), exhibits excellent performance in both detection accuracy and efficiency. It achieves an mAP@0.5 of 91.8%, surpassing YOLOv5s, YOLOv7t, Faster-RCNN, MobileNetv3-YOLO, and the baseline YOLOv8n model. The inference speed reaches 41.6 frames per second, significantly exceeding that of other typical models. The number of parameters (3.0 M) and computational cost (8.3 GFLOPs) remain lightweight. Although there is a slight decrease in precision compared to YOLOv8n, the recall rate is substantially improved by 4.9 percentage points, achieving a better balance between precision and recall. The model demonstrates the best overall performance, effectively validating the effectiveness of its structural optimization.

### 3.8 Performance comparison of optimal models on the independent test set

To comprehensively evaluate the generalization capability of the proposed improved YOLOv8n model, this study further conducted external validation experiments on the publicly available UR Fall dataset. The results were compared with those obtained on the self-constructed dataset to examine the model’s detection performance under unseen scenarios, varying lighting conditions, and diverse fall postures. The results are presented in Table 9.

**Table 9:** Comparative experiments on different datasets.

Model design	Dataset	Precision	Recall	mAP@0.5	Parameters (M)	GFLOPs	FPS
YOLOv8n	UR Fall Dataset	85.1%	89.7%	92.1%	3.0	8.1	23.5
YOLOv8n-ours		87.1%	85.7%	92.3%	3.0	8.3	39.1
YOLOv8n	Our Fall Dataset	913%	78.9%	89.7%	3.0	8.1	28.7
YOLOv8n-ours		86.6%	83.8%	91.8%	3.0	8.3	41.6

The experimental results demonstrate that the improved model, YOLOv8n-ours (YOLOv8n-C2f_PKI + SimSPPF), while maintaining the same parameter count (both at 3.0 M), achieves improvements in both mean Average Precision (mAP@0.5) and inference speed (FPS) compared to the original YOLOv8n across both datasets. On the UR Fall dataset, mAP@0.5 increases from 92.1 to 92.3%, and FPS rises from 23.5 to 39.1. On the self-constructed dataset, mAP@0.5 significantly improves from 89.7 to 91.8%, and FPS increases from 28.7 to 41.6. Although the computational complexity (GFLOPs) increases slightly, the model exhibits data-dependent characteristics in the trade-off between precision and recall: on the UR Fall dataset, the improved model places greater emphasis on detection precision (with a 2.0% increase in Precision) at the cost of a slight decrease in recall; on the self-constructed dataset, however, it prioritizes recall (with a 4.9% increase in Recall), accompanied by a corresponding reduction in precision. This demonstrates the adaptability of the improved modules to data with different distributions. Overall, the proposed improved model exhibits excellent detection performance and model generalization capability on both the publicly available UR Fall dataset and the self-constructed dataset, where the slight increase in GFLOPs did not compromise the actual inference speed.

### 3.9 Human fall detection results

**Figure 9:**
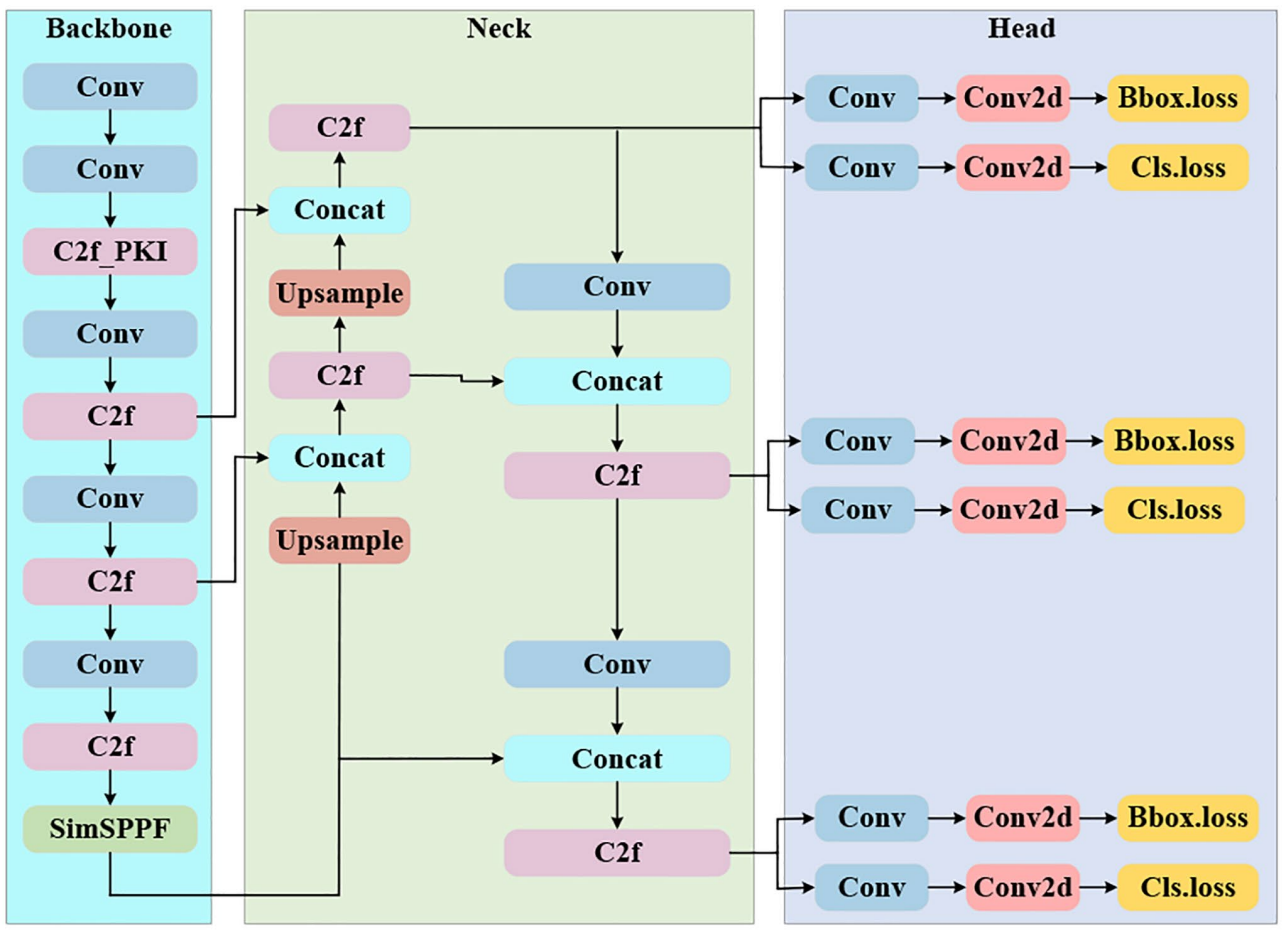
The network architecture of the optimized YOLOv8n model, where two core modules in the original framework are replaced: the C2f module in the backbone is updated to C2f_PKI, and the SPPF module is replaced with SimSPPF.

Based on the above 20 sets of experimental results, it was ultimately confirmed that the original YOLOv8n model, after integrating the C2f_PKI and SimSPPF modules, achieved the best overall detection performance, with an mAP@0.5 value of 91.8% on the dataset. The network structure diagram of the best model is shown in Figure 9.

**Figure 10:**
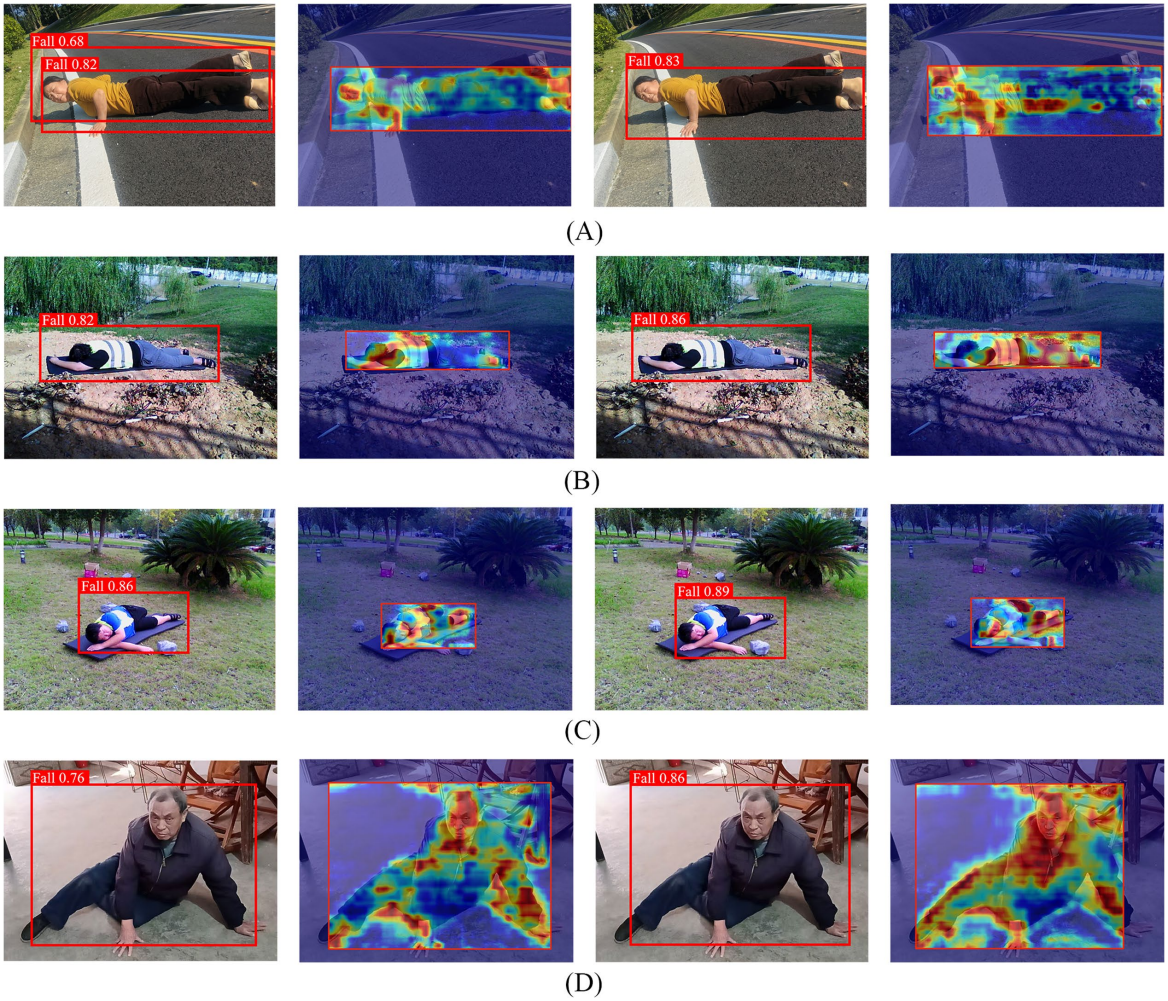
Performance comparison of fall detection models. This figure presents the fall detection results (including detection boxes with confidence scores and Grad-CAM heatmaps) of the YOLOv8n baseline model (left two columns) and the improved YOLOv8n-C2f_PKI + SimSPPF model (right two columns) across four scenarios: **(A)** Corresponds to the first row (4 images total), representing a high-noon highway scene under intense sunlight (13:00); **(B)** corresponds to the second row (4 images total), representing a late-afternoon park scene with pronounced shadows (16:00); **(C)** corresponds to the third row (4 images total), representing a low-light overcast scene with small objects; **(D)** corresponds to the fourth row (4 images total), representing a cluttered indoor living room scene.

To verify the on-site detection performance of the best model, this paper selected four real-life scenes with different light and shadow intensities, including a highway at 1 p.m. with strong light, a park at 4 p.m. with prominent light and shadow, a small target in weak light on a cloudy day, and a living room with a complex home background. The best model was compared with the baseline model YOLOv8n in a horizontal manner. To further reveal the areas of concern in the model’s decision-making process, the Grad-CAM (32) technique was used to generate heat maps, visually presenting the model’s response to key feature areas. The actual detection results are shown in Figure 10.

The results show that the YOLOv8n baseline model performs well overall, but still has detection deviations in some scenarios. As shown in Figure 10, the YOLOv8n baseline model has double detection boxes. In contrast, the dual-module model incorporating C2f_PKI and SimSPPF outperforms the baseline model in both target box selection and detection confidence values in all actual detection groups. Particularly in group (D), the confidence level has been significantly improved by 10 percentage points. Additionally, by comparing the Grad-CAM heatmaps of the original YOLOv8n and the improved model, the improved model demonstrates better focusing ability on key part information.

## 4 Conclusion

To address the challenges of low detection accuracy and high difficulty in detecting falls of the older adult(s) in complex backgrounds, this study proposes a fall detection method for the older adult(s) based on the improved YOLOv8n. Firstly, a high-quality multi-pose human fall database covering different light intensities and various real scenarios was constructed. Secondly, based on the YOLOv8n model, seven advanced lightweight and attention mechanism modules, including RepVGG, SPPF_LSKA, SimSPPF, SEAttention, ShuffleAttention, C2f_ScConv, and C2f_PKI, were introduced. A total of 20 groups of fusion improvement experiments were conducted, including single-module, dual-module, and triple-module combinations, to systematically analyze the impact of each module on the model’s performance. Additionally, four mainstream models and the publicly available UR Fall dataset were introduced as experimental controls. The experimental results demonstrate that the dual-module model incorporating C2f_PKI and SimSPPF achieves the best overall performance, attaining an mAP@0.5 of 91.8% on the self-constructed dataset. Under the condition of maintaining the same parameter count, this model improves the mAP@0.5 by 2.1% compared to the original YOLOv8n, while reaching an FPS of 41.6. Both its detection accuracy and efficiency surpass those of mainstream models such as YOLOv5s and Faster-RCNN. In real-world scenarios with complex lighting conditions, its detection accuracy outperforms the original model by up to 10 percentage points.

However, given the complexity of fall detection environments and the diversity of improvement strategies, this study still has certain limitations. Due to constraints in computational resources, it was not possible to exhaustively explore all seven possible combinations of modules. Future work could combine theoretical analysis with systematic ablation experiments to further investigate multi-module integration strategies and embedding mechanisms based on the existing efficient modules. This would facilitate the construction of fall detection models with higher performance and stronger interpretability, thereby promoting the practical application of related algorithms in real-world complex scenarios.

## Data Availability

The raw data supporting the conclusions of this article will be made available by the authors, without undue reservation.
