# Correction: Research on fall detection algorithm for older adults in complex lighting environments based on improved YOLOv8n

**DOI:** 10.3389/fpubh.2026.1839054

**Published:** 2026-04-07

**Authors:** Xiaojin Gan, Yihui Lai, Gui Xiao, Yuting Xiong, Leyan Yu, Xiaoling Zhou, Songying Wu

**Affiliations:** 1 Department of Gynecology, The Affiliated Hospital of Jiangxi University of Chinese Medicine, Nanchang, Jiangxi, China; 2 School of Intelligent Medicine and Information Engineering, Jiangxi University of Chinese Medicine, Nanchang, Jiangxi, China; 3 Department of Nursing, Jiangxi University of Chinese Medicine, Nanchang, Jiangxi, China

**Keywords:** attention mechanism, fall detection, lightweight, module fusion improvement, YOLOv8n

The affiliation “Department of Gynecology, The Affiliated Hospital of Jiangxi University of Traditional Chinese Medicine, Nanchang, Jiangxi, China” was erroneously given as the affiliation for author Xiaojin Gan. The correct affiliation is “Department of Gynecology, The Affiliated Hospital of Jiangxi University of Chinese Medicine, Nanchang, Jiangxi, China”.

In the **Acknowledgements**, “The Affiliated Hospital of Jiangxi University of Traditional Chinese Medicine - Gynecology Department” should appear as “Department of Gynecology, The Affiliated Hospital of Jiangxi University of Chinese Medicine”.

The original version of this article has been updated.

